# Intensity of African Humid Periods Estimated from Saharan Dust Fluxes

**DOI:** 10.1371/journal.pone.0170989

**Published:** 2017-01-27

**Authors:** Werner Ehrmann, Gerhard Schmiedl, Sarah Beuscher, Stefan Krüger

**Affiliations:** 1 Institut für Geophysik und Geologie, Universität Leipzig, Talstraße 35, Leipzig, Germany; 2 Centrum für Erdsystemforschung und Nachhaltigkeit, Universität Hamburg, Bundesstraße 55, Hamburg, Germany; University of California San Diego, UNITED STATES

## Abstract

North Africa experienced dramatic changes in hydrology and vegetation during the late Quaternary driven by insolation-induced shifts of the tropical rain belt and further modulated by millennial-scale droughts and vegetation-climate feedbacks. While most past proxy and modelling studies concentrated on the temporal and spatial dynamics of the last African humid period, little is known about the intensities and characteristics of pre-Holocene humid periods. Here we present a high-resolution record of fine-grained eastern Saharan dust from the Eastern Mediterranean Sea spanning the last 180 kyr, which is based on the clay mineral composition of the marine sediments, especially the kaolinite/chlorite ratio. Minimum aeolian kaolinite transport occurred during the African Humid Periods because kaolinite deflation was hampered by increased humidity and vegetation cover. Instead, kaolinite weathering from kaolinite-bearing Cenozoic rocks was stored in lake basins, river beds and soils during these periods. During the subsequent dry phases, fine-grained dust was mobilised from the desiccated lakes, rivers and soils resulting in maximum aeolian uptake and transport of kaolinite. The kaolinite transport decreased again when these sediment sources exhausted. We conclude that the amount of clay-sized dust blown out of the Sahara into the Eastern Mediterranean Sea is proportional to the intensity of the kaolinite weathering and accumulation in soils and lake sediments, and thus to the strength of the preceding humid period. These humid periods provided the windows for the migration of modern humans out of Africa, as postulated previously. The strongest humid period occurred during the Eemian and was followed by two weaker phases centred at ca. 100 ka and ca. 80 ka.

## Introduction

The North African environments underwent major changes in the intensity of precipitation during late Quaternary time. These changes include both monsoon-paced pluvials [1, 2] of the African Humid Periods (AHPs) and millennial-scale droughts that were induced by cold intervals of the northern high-latitudes and occurred during the last glacial period [3]. Proxy data and model results indicate a differential hydrological response in the western *versus* eastern parts of North Africa [4–6]. To date, there is little knowledge of the intensities and characteristics of pre-Holocene humid periods. There is an on-going debate about abrupt versus gradual transitions between the AHPs and the subsequent dry phases, which likely involved significant vegetation-climate feedbacks and non-linear ecosystem responses [2, 7–9].

**Fig 1:**
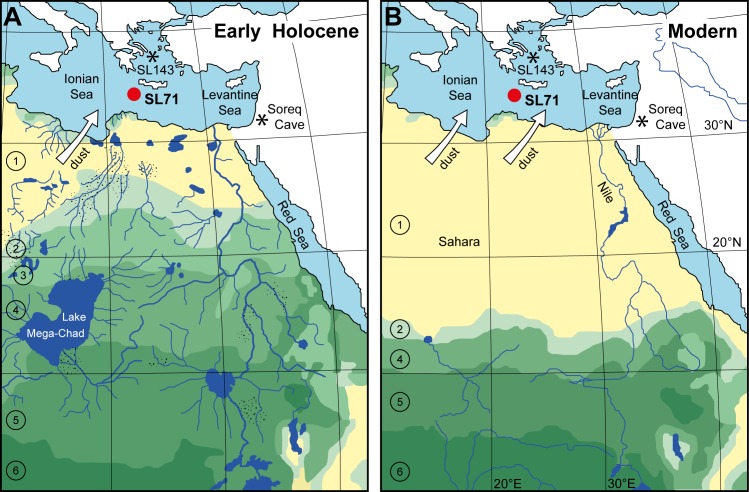
Location and North African vegetation and drainage changes between humid and arid phases. (A) Simulated biome distribution (simplified after [4]) and North African drainage pattern (simplified after [11]; no data for the southwest part of the map) during the early Holocene humid phase. (B) Biome distribution at present-day (simplified after [4]). 1: hot desert; 2: warm grass/shrub; 3: temperate woods/shrub; 4: tropical bush/savannah; 5: tropical dry forest/savannah; 6: tropical forest. Stippled areas: alluvial fans. Red dot: position of the investigated sediment core M40/4_SL71 in the EMS; asterisks: positions of marine sediment core M51/3_SL143 and speleothem Soreq Cave mentioned in the text.

The AHPs were characterised by numerous large lakes and rivers in present-day arid regions (Fig 1) [10–13], enhanced river runoff [14, 15], dense vegetation cover (Fig 1) [4, 5, 8] and reduced erosion and transport by wind [7, 16]. In the Eastern Mediterranean Sea (EMS), enhanced river runoff during AHPs ultimately led to a shutdown of deep-water formation documented in the cyclic deposition of sapropel layers [17].

The strong climatic and environmental changes also controlled the pathway, transport processes and source areas of the terrigenous sediment particles of the EMS sediments. In particular, clay mineral associations proved to be good proxies for riverine suspended load and fine-grained Saharan dust. The latter is documented by a high abundance of kaolinite (e.g. [18]). These strong signals are expressed in the kaolinite/chlorite (K/C) ratio [16], which is the focus of this paper.

**Fig 2:**
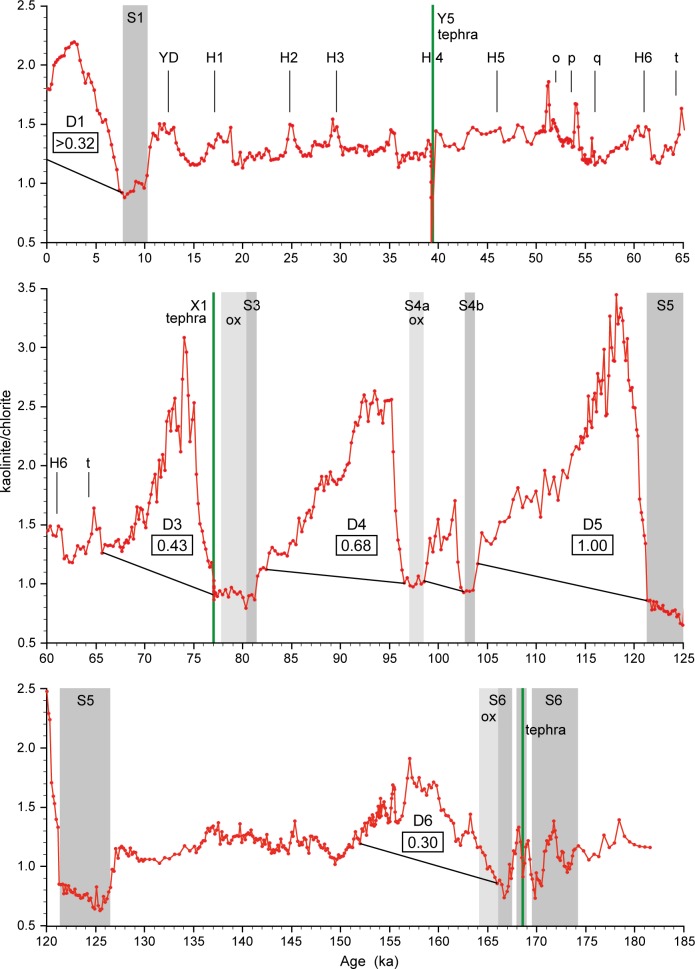
Saharan dust record of core M40/4_SL71 from the Ionian Sea. The dust record is expressed as kaolinite/chlorite ratio, and the dust index (framed numbers) of the dust pulses D1 to D6 is calculated from the kaolinite/chlorite peak areas. Sapropels S1, S3, S4, S5 and S6 are indicated by grey bars, and oxidised sapropels by light grey bars. Tephra layers are shown as green lines. Younger Dryas (YD), Heinrich Events (H) and other cold events of the North Atlantic and Arctic regions (o–t) are labelled [19]. MIS = Marine Isotope Stage.

We present a continuous high-resolution 180 kyr K/C record from sediments of core M40/4_SL71 from SW of Crete (Figs 1 and 2) in order to reconstruct the amount of fine-grained Saharan dust transported to the EMS. According to our hypothesis, kaolinite-bearing weathering and erosion products accumulated in lakes, river beds and soils during humid periods. This material was prone to aeolian uptake when arid conditions were established. Therefore, the dust pulses should be proportional to the intensity of preceding AHPs and associated African monsoon maxima. Our data also provide information on the abruptness of AHP terminations in the dust source areas.

### Origin of Kaolinite in EMS Sediments

Kaolinite found in the EMS has its source almost exclusively in North Africa [16, 18, 20]. It originates from the erosion of lateritic soils and kaolinite-rich sedimentary rocks that have a Mesozoic to Paleogene age. The clay fractions of these sedimentary rocks have kaolinite concentrations of up to 70% [21, 22]. The kaolinite occurrence in Quaternary lake sediments and soils [23, 24] demonstrates that parts of the erosion products were stored in such deposits during humid periods.

The Nile River delivers kaolinite from the African continent into the EMS [15, 20, 21, 25]. However, the Nile suspended load does not reach the site of the studied sediment core M40/4_SL71 SW of Crete [20, 26, 27]. Because the site is relatively close to the African continent, it receives a substantial amount of aeolian sediment from the Sahara. The Scirocco winds blow from the south and transport large amounts of dust, mainly during short events in spring and early summer [28–32]. Today, the mean dust accumulation just north of M40/4_SL71, on the island of Crete, is 10–100 g/m^2^yr [28, 33]. The most important present-day sources for dust reaching the EMS are probably the eastern and central Libyan deserts and southern Algeria [28–31]. Kaolinite is a common mineral in North African dust and is most abundant in dust originating from the south and central Sahara, with reported concentrations of up to 50% [29, 34, 35].

Thus, kaolinite is derived from the erosion of outcropping kaolinite-rich sedimentary rocks and, more importantly, of their recycled products stored in lake sediments and soils. Vegetation-free, dry lake beds in arid regions are the preferential areas for the entrainment of fine-grained dust in several deserts [36–38]. Other quantitatively important dust sources such as sand dune fields and alluvial fans mainly provide coarser grained quartz particles, but fewer clay minerals. It is well established that these dust fluxes are predominantly controlled by wind and topography, rather than by aridity (e.g. [39–41]). More details on dust generation and transport are described elsewhere (e.g. [30–32, 38, 42]).

In addition to kaolinite, palygorskite is a typical wind-blown mineral in EMS sediments and an excellent tracer of Saharan dust. It mainly derives from Palaeogene North African deposits occurring from Morocco and Tunisia to Libya and Egypt [18, 29, 32, 43]. As for kaolinite, we assume that palygorskite sources experience intense erosion during humid periods and the weathering products are subsequently stored in depressions, lake and river sediments and soils.

## Material and Methods

The studied sediment core M40/4_SL71 was retrieved in 1998 from the SE Ionian Sea, ca. 50 km SW of Crete (Fig 1; 34°48.67’ N, 23°11.65’ E, 2788 m water depth) during cruise M40/4 of the German research vessel “Meteor”. Core retrieval was carried out in compliance with international law and regulations of the neighbouring countries as mediated by the “Control Station German Research Vessels” (formerly “METEOR Operations Control Office”) of the University of Hamburg. The sediments consist mainly of yellowish, brownish and greyish foraminifera-nannofossil ooze [44]. Five sapropel layers occur within the sequence (Fig 2) and are characterised by their olive grey to olive black colour indicating preserved organic material, yellowish colour indicating post-sedimentary oxidation, and/or laminations. The sapropels are S1 (15–19 cm), S3 (182–184 cm, oxidised 178–182 cm), S4a (215.5–219 cm, oxidised), S4b (227–228 cm), S5 (261.5–277 cm), and S6 (387–414.5 cm, oxidised 384.5–387 cm). The latter shows two interruptions at 391–392 cm and 395.5–397 cm. Furthermore, three volcanic ash layers were recovered. The upper one at 84–88 cm represents the Y5-Tephra. The middle one at 173–176 cm occurs shortly above sapropel S3 and probably can be assigned to the X1-Tephra (Y9; cf. [45]). The lower one occurs within S6 at 394.5 cm and therefore could be identified as the V1-Tephra [46].

We sampled and analysed the upper 421 cm of the core with a spacing of 0.5 cm. We removed carbonate and organic carbon and then isolated the clay fraction (<2 μm). Texturally oriented aggregates were produced by rapidly filtering the clay suspension through a membrane filter of 0.15 μm pore width and mounted onto aluminium slides. The slides were X-rayed with CoKα radiation (30 kV; 15 mA) in the range 27.5–30.6° 2ϴ with a step size of 0.01° 2ϴ and a measuring time of 4 sec/step. The K/C ratio was calculated based on the (002) kaolinite and (004) chlorite peak areas.

**Fig 3:**
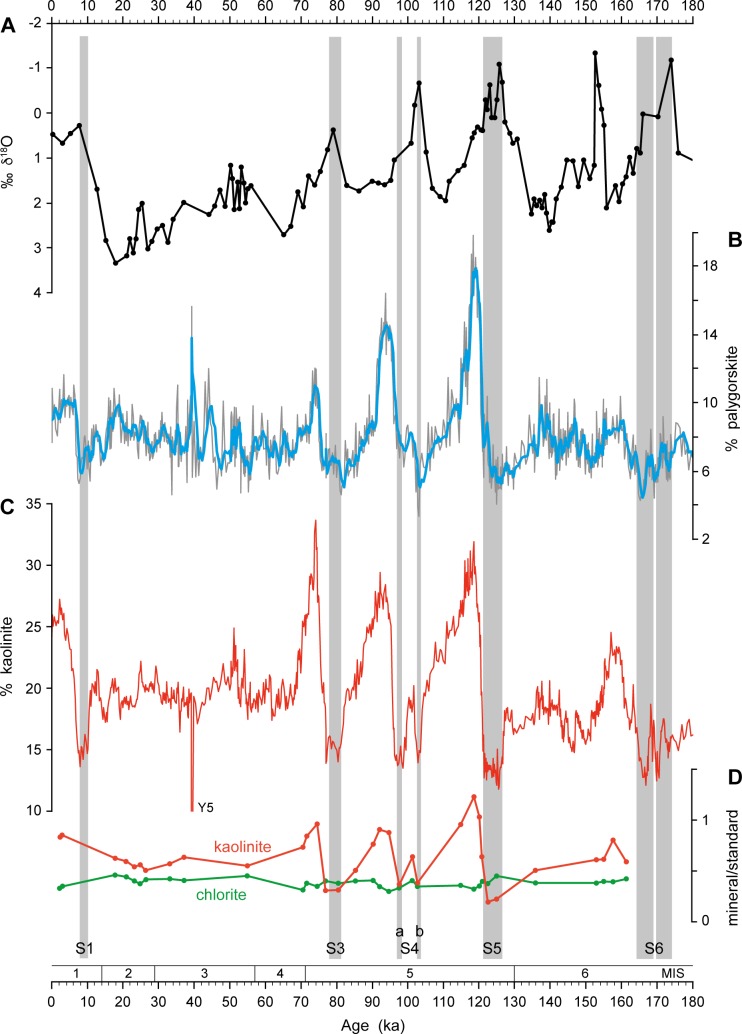
Additional data for sediment core M40/4_SL71. (A) Oxygen isotope record (data from [50]). (B) Concentration of palygorskite within the clay mineral assemblage, including 5-point running average. (C) Concentration of kaolinite within the clay mineral assemblage. Grey bars indicate sapropel layers. (D) Ratios of kaolinite/standard (red) and chlorite/standard (green).

In addition to the calculation of the K/C ratio (Fig 2), we also performed standard clay mineral analyses (e.g. [47]). The clay mounts were solvated with ethylene-glycol vapour and X-rayed in the range 3–40° 2ϴ (step size 0.02° 2ϴ, measuring time 2 s/step). We calculated the percentages of smectite, illite, chlorite, palygorskite and kaolinite based on their peak areas and empirically estimated weighting factors [48, 49]. The percentages of kaolinite and palygorskite are shown in Fig 3.

Next, we added to a representative set of carbonate-free clay samples a constant proportion (10%) of an internal standard consisting of calcite, and then calculated the kaolinite/standard and chlorite/standard peak area ratios using the 3.58 Å peak of kaolinite, the 3.54 Å peak of chlorite and the 3.04 Å peak of calcite (Fig 3).

The dust pulses D1 and D3–D6 have been numbered according to the preceding sapropels. We calculated a dust index for the individual pulses, based on an integration of the area enclosed between the K/C curve and the base line (Fig 2). The maximum value of the Eemian was set as 1.0 and the other maxima were related to this value level.

### Age Model

We based the age model for core M40/4_SL71 on a graphic correlation of the published stable oxygen isotope record of *Globigerinoides ruber* (white) ([50]; Fig 3) with the LR04 benthic oxygen isotope stack [51], aided by the software AnalySeries 2.0 [52]. The LR04 stack has an uncertainty of 4 kyr.

The age model was refined by five ^14^C-accelator mass spectrometry (AMS) dates performed by Beta Analytic Radiocarbon Dating Laboratory (Table 1). For the ^14^C-dating we used well-preserved surface-dwelling planktonic foraminifera (*G*. *ruber*, *G*. *bulloides*, *G*. *sacculifer*, *O*. *universa*) that represent the age of the surface waters. We applied an eastern Levantine Sea Delta-R of 3 ± 66 years (Marine Reservoir Database). The radiocarbon ages were converted to calendar years using the Marine13 data base [53].

**Table 1:** Data used for constructing the age model for the investigated sediment core M40/4_SL71.

Depth (cm)	Age (cal ka BP)	Datum
0.00	0.000	sediment surface
2.50	0.2	δ^18^O record
4.75	1.935 ± 0.165	AMS^14^C
13.25	6.865 ± 0.165	AMS^14^C
15.00	7.8	δ^18^O record
20.25	10.825 ± 0.265	AMS^14^C
35.00	17.9	δ^18^O record
36.25	18.790 ± 0.120	AMS^14^C
50.25	25.540 ± 0.390	AMS^14^C
84.00	39.280 ± 0.110	Top Y5-Tephra
88.00	39.280 ± 0.110	Base Y5-Tephra
100.00	50.2	δ^18^O record
127.50	56.1	δ^18^O record
147.50	67.2	δ^18^O record
157.50	70.7	δ^18^O record
177.50	77.5	δ^18^O record
192.50	86.5	δ^18^O record
215.50	96.4	δ^18^O record
227.50	103.3	δ^18^O record
239.50	114.3	δ^18^O record
270.34	124.0	δ^18^O record
286.80	130.0	δ^18^O record
291.73	135.1	δ^18^O record
305.55	139.9	δ^18^O record
346.79	153.0	δ^18^O record
366.48	155.9	δ^18^O record
388.83	167.0	δ^18^O record
414.47	174.0	δ^18^O record
425.08	185.0	δ^18^O record
465.49	192.0	δ^18^O record

Age points are derived from AMS ^14^C dates, the age of the Y5-Tephra [54] and graphical correlation of the δ^18^O record of *G*. *ruber* white [50] with the LR04 stack [51].

A further age control point came from the Y5-Tephra (39.280 ± 0.110 ka [54]). We did not use the X1 and V1 tephras as age markers, because of their poorly constrained ages of 75.3 ± 3.0 ka [45, 55] and ca. 170 ka [56], respectively. According to our age model, X1 has an age of 77.05 ka, and V1 an age of 168.60 ka, confirming the validity of the model.

The investigated core section has a basal age of about 180 ka. Average linear sedimentation rates are ca. 2.3 cm/kyr. Thus, average time resolution for a 0.5 cm sampling interval is ca. 220 years.

A major distortion of environmental signals in our core by bioturbation can be excluded. Abyssal benthic ecosystems of the EMS are characterized by oligotrophic to ultra-oligotrophic (particularly during interglacials) conditions as evidenced from benthic foraminiferal faunal successions [57]. These conditions only support low-density and low-diversity benthic communities almost exclusively composed of epifaunal and very shallow infaunal organisms during interglacials [58]. The lack of deeper infaunal organisms likely results in strongly reduced bioturbation rates when compared to other more food-enriched deep-sea environments.

## Results

The results of the clay mineral analyses on sediments of core M40/4_SL71 are presented in Figs 2 and 3, which also show the position of the sapropel and tephra layers. The raw data presented in this paper are available at https://doi.pangaea.de/10.1594/PANGAEA.869095.

The typical features in the K/C curve are a minimum of 0.7 to 0.9 during sapropel deposition and a sharp and drastic increase thereafter to values of up to 3.5, followed by an almost exponential decrease to a base level. A series of smaller K/C maxima occurs during the last and penultimate glacial intervals (Fig 2).

The similarity between the palygorskite concentration, the kaolinite concentration and the K/C ratio (Figs 2 and 3) strongly supports a common geographic origin of the two wind-blown minerals kaolinite and palygorskite, likely in Algeria, Tunisia and Libya. The Bodélé depression (Lake Mega-Chad; Fig 1), the most intense and persistent dust source world-wide [30, 40], is an unlikely source because palygorskite has not been described so far in dust originating from there [32, 43].

The palygorskite concentrations fluctuate much more than the K/C record. This, however, is due to some methodological problems. Palygorskite is difficult to quantify, because its 10.5 Å main reflection does not form an isolated peak in the M40/4_SL71 XRD records, but is expressed as a shoulder on the much larger 10 Å illite peak.

The curve of kaolinite percentages through time (Fig 3) is almost identical to the K/C curve (Fig 2). Nevertheless, we prefer to present the K/C ratio rather than kaolinite percentages as a proxy for Saharan dust influx to the EMS. Using K/C has the advantage that it largely avoids dilution effects caused by the closed sum system, with percentages of the clay minerals smectite, illite, chlorite, kaolinite and palygorskite adding up to 100%. In such a system, a change in the amount of one clay mineral, especially of the abundant smectite or illite, must always cause changes in the concentrations of the others.

To discern whether kaolinite or chlorite is the driver of changes in the K/C ratio, we measured them against an internal standard. Kaolinite/standard and chlorite/standard ratios give kaolinite and chlorite abundances that are independent from each other and from the concentrations of other minerals. Our data show that the chlorite/standard ratio is almost constant throughout the core, and fluctuates only between 0.30 and 0.46. In contrast, the kaolinite/standard ratio shows major fluctuations between 0.20 and 1.23 and reproduces the K/C curve (Fig 3). Thus, the fluctuations in the K/C ratio in core M40/4_SL71 are caused almost exclusively by changes in the kaolinite abundance and not by dilution effects. This is confirmed by the distribution of chlorite in the seafloor surface sediments of the EMS. Chlorite is ubiquitous in minor amounts in EMS sediments, but no distinct source for this mineral is evident in the hinterland [20, 25, 59]. Also, late Quaternary sediment records of the EMS show only minor fluctuations in chlorite concentrations [15, 25, 47]. Accordingly, the K/C ratio is a robust proxy for dust influx from the North African deserts into the EMS, and is corroborated by its good correlation with the palygorskite abundance.

## Discussion

### African Humid Periods

**Fig 4:**
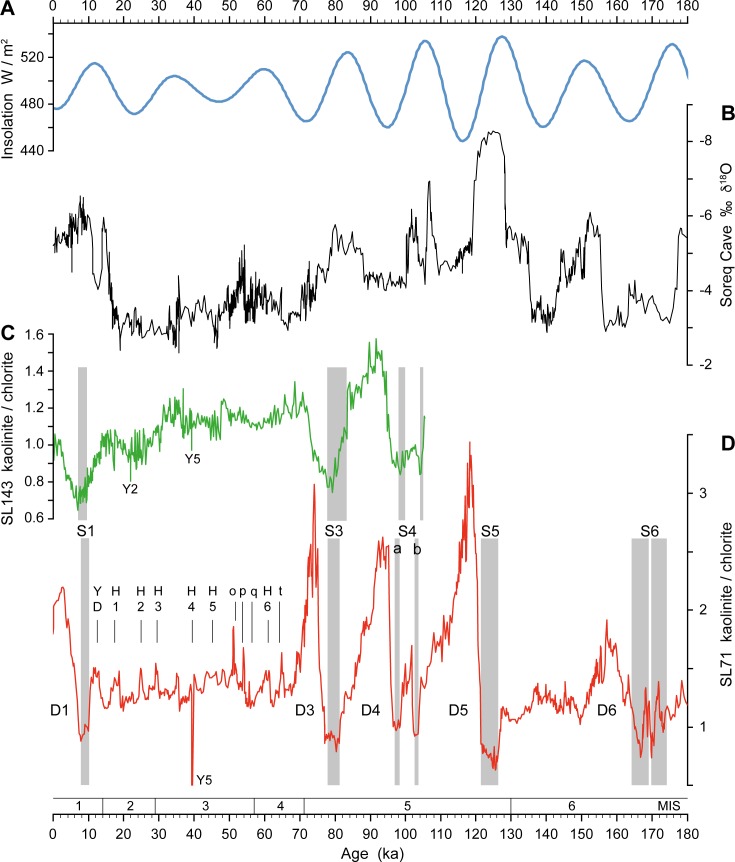
Correlation of M40/4_SL71 dust record with other proxy climate data. (A) June insolation at 30°N [62]. (B) Speleothem δ^18^O record of Soreq Cave, Israel [63]. (C) Saharan dust record from core M51/3_SL143 in the Aegean Sea documented by the kaolinite/chlorite ratio [16]. (D) Saharan dust record from core M40/4_SL71 in the Ionian Sea documented by the kaolinite/chlorite ratio, with dust pulses D1 to D6 (for high resolution see Fig 2). Grey bars indicate sapropel layers (S). Younger Dryas (YD), Heinrich Events (H) and other cold events (o–t) are labelled [19]. MIS = Marine Isotope Stage.

Our M40/4_SL71 K/C record from the EMS shows low values during the AHPs with minima confined to the times of sapropel formation (Figs 2 and 4). During these pluvial periods large amounts of kaolinite were provided by the weathering and erosion of older kaolinite-bearing sedimentary rocks and incorporated into North African soils, alluvial fans and lake sediments. However, the humidity and the accompanying vegetation cover [60, 61] hampered wind-erosion and thus kaolinite transport to the EMS [16, 38].

**Fig 5:**
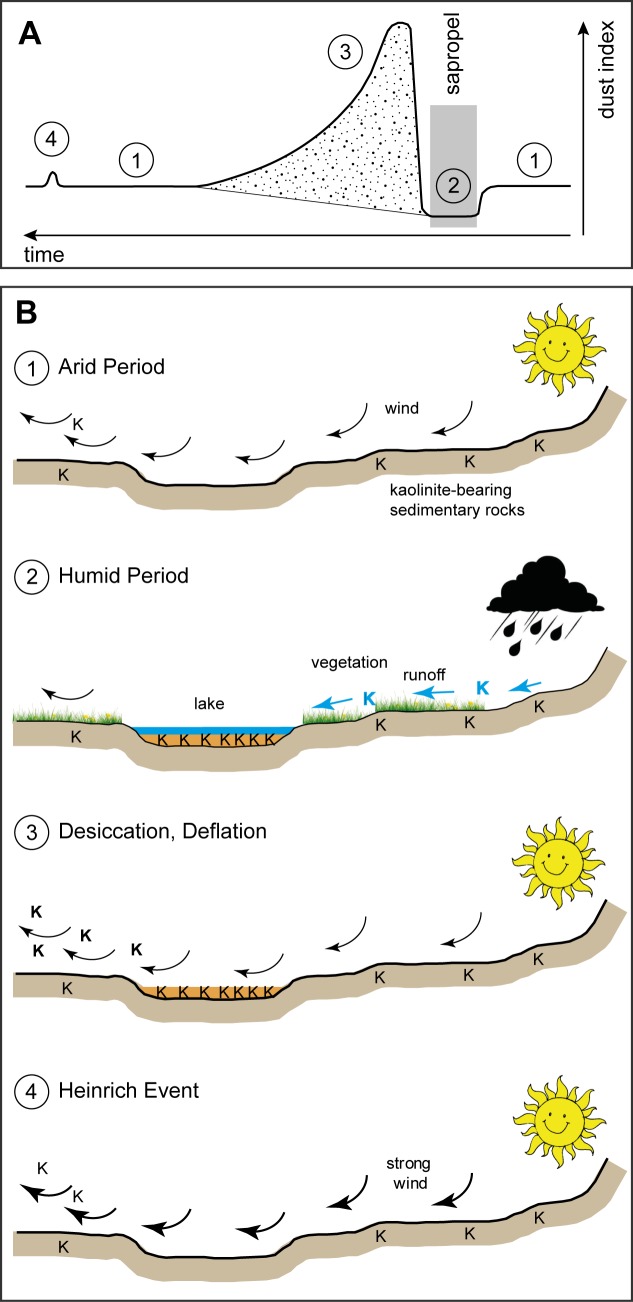
Conceptual model of dust dynamics. (A) Changes in the quantity of fine-grained dust influx to the EMS with time. 1: Background level; 2: Pluvial phase with sapropel formation; 3: Dust phase, 4: Glacial drought. The dotted area was used to calculate the dust index. (B) Sketches for the individual phases labelled in panel (A). 1: Arid periods with restricted deflation of kaolinite from kaolinite-bearing sedimentary rocks. 2: Cessation of aeolian transport of kaolinite during humid periods due to vegetation and reduced wind activity. Clay-rich erosion products accumulate in lake basins. 3: With the return of arid conditions, the kaolinite-rich lake sediments are blown out from the desiccated basins leading to maximum kaolinite influx to the Eastern Mediterranean Sea. 4: Intensified aeolian activity during glacial droughts (Heinrich Events) causes minor kaolinite maxima.

All terminations of the sapropel formation phases in the EMS were followed by a strong increase in the K/C ratio. With the establishment of arid conditions, weathering and sediment production decreased and accumulation in Saharan lakes and soils stopped. The lakes desiccated after the pluvial periods and the vegetation retreated. Therefore, the palaeo-lake beds and soils with kaolinite stored in them became prone to wind erosion, leading to a strongly enhanced availability for dust uptake and transport, and thus to the dust pulses D1 through D6 in the EMS (Fig 5).

The gradual decline of the K/C ratio after the maximum of each major dust phase mainly reflects the shrinking area of the desiccated lakes and soil-covered areas and thus a decrease in the amount of material that can easily be taken up by wind. The dust phases lasted for approximately 11–19 kyr (Table 2). The relatively high modern K/C ratio indicates that the Holocene dust phase has not yet come to an end. This is corroborated by remnants of lake sediments in the present-day Sahara (e.g. [40, 41, 64]).

**Table 2:** Correlation of dust influx phases D1, D3–D6 with insolation maxima [62] and monsoon index [1] during the preceding pluvial phases with formation of sapropels S1, S3–S6.

Dust phase	Duration (ka)	Dust index	Sapropel	Insolation Maximum (W/m^2^)	Monsoon Index
**D1**	>7.7	>0.32			
** **			**S1**	515	42
**D3**	11.4	0.43			
** **			**S3**	524	43
**D4a**	15.0	0.68			
**D4b**	4.0				
** **			**S4**	534	49
**D5**	17.3	1.00			
** **			**S5**	537	60
**D6**	14.0	0.30			
			**S6**	539	56

Our K/C data, aided by the palygorskite data, do not represent the total dust flux but rather the proportion of clay minerals in fine-grained dust derived mainly from lake sediments and soils. A similar Saharan dust record is reported from the Aegean Sea sediment core M51/3_SL143 [16], but is of lower amplitude due to the more distal position of the site (Figs 1 and 4). Corresponding records come from the Nile sediment discharge and reflect enhanced rainfall during AHPs in the Ethiopian Highlands, which are the Nile headwaters [14, 15]. Evidence for alternating humid and dry phases is also derived from lake-level fluctuations in tropical Africa. Despite dating uncertainties and recurrent desiccation phases, the lake-level records reflect a pattern of humid phases and associated high lake levels during monsoon maxima and subsequent dry phases associated with lake-level drops [65–67].

The gradient of the K/C curve can be used as an indicator for the relative abruptness of the end of an AHP (Fig 2). We expect that the gradient is not modulated by bioturbation rates, because we can assume analogous environmental conditions and therefore similar bioturbation rates during all humid/dry transitions. Neither is the gradient substantially modulated by sedimentation rates, because they are similar during all transitions (ca. 2–3 cm/kyr). The K/C ratio increased abruptly within ca. 1.5–2.0 kyr after sapropels S5 (gradient 1.1/ kyr), S4 (1.0/ kyr) and S3 (0.8/ kyr), whereas the increase took much longer and was more gradual after S6 (6.5 ka; 0.15/ kyr) and S1 (5 ka; 0.25/ kyr). This suggests that the strength of the climate-vegetation feedback in the catchment areas was variable during the terminations of the individual pluvial phases. Especially strong feedbacks and resulting abrupt shifts in the eastern Saharan ecosystem state are interpreted for the Eemian period.

Glacial boundary conditions in North Africa were generally cool and dry with expansion of desert areas and savannahs into areas covered by forests at present-day [10, 61]. Also during sapropel S6 formation, the monsoon was likely less intense than during interglacial sapropel events [68]. Our data across S6 furthermore indicate strongly fluctuating dust fluxes on centennial to millennial scales (Figs 2–4). They suggest the presence of a less sustained and more variable vegetation cover, and the importance of millennial-scale climate variability under the influence of glacial boundary conditions similar to those observed during the last glacial period.

A more gradual transition from wet to dry conditions is also observed for the Holocene, which is consistent with observations from palynological and sedimentological data [8, 12, 69]. Contrasting results, however, come from dust records of western North Africa [7] and from model experiments [70, 71]. These discrepancies may be explained by different vegetation responses to humidity between eastern and western North Africa [4, 5], although the processes responsible for this asymmetry are not yet fully understood.

### Dust Index

We calculated a dust index in order to quantify the influx of fine-grained Saharan dust during the desiccation phases. The dust index is a proxy for the volume of deflateable weathering products that have been produced during the preceding AHP, which in turn is a proxy for the intensity of the AHP, i.e. the degree of humidity and the temporal extent of the AHP.

**Fig 6:**
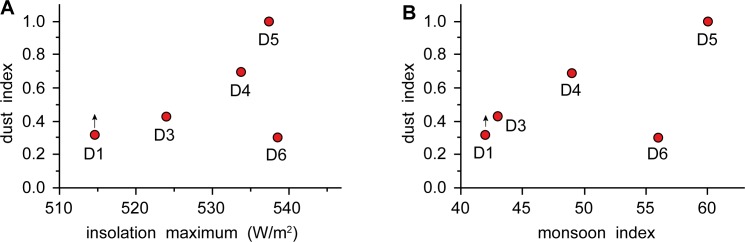
Correlation of the dust index with insolation maxima and monsoon index. The dust index of the influx pulses D1, D3, D4, D5 and D6 in core M40/4_SL71 is correlated with (A), the insolation maximum [62] and (B), the monsoon index of the preceding pluvial phase [1]. The calculated dust index for phase D1 is a minimum value, because this phase is still active.

The dust index correlates to both preceding insolation maxima and the monsoon index, which is based on the difference in insolation between the equator and the northern tropics [1]. Except for D6, there is an almost linear relationship between dust index and monsoon index (Fig 6; Table 2). This implies that the stronger the North African monsoon the more erosion products accumulated during the pluvial phases in lakes, soils and depressions and were blown out after their termination. Thus, the amount of fine-grained dust found in our core from the EMS is not a function of wind strength but of availability of material that can be entrained in the atmosphere.

The dust index implies a decreasing intensity of the pluvial phases with sapropels S5 (dust index 1.0), S4 (0.68), S3 (0.43), S1 (>0.32), and S6 (0.30). The exceptional strength of the pluvial phase connected to Eemian sapropel S5 is also reflected in the well-developed fossil river/wadi systems and postulated lake systems in North Africa and the Arabian Peninsula [11, 13, 72, 73]. There is increasing evidence that the establishment of palaeohydrological networks and corridors during the Eemian initiated the migration of modern humans across the formerly hyper-arid areas of North Africa and Arabia [73–76]. In addition, the speleothem record of Soreq cave ([63]; Fig 4) shows highest rainfalls in the eastern Mediterranean during this time. The Soreq data also exhibit a two-phase humid period associated with S4, which in M40/4_SL71 is split into two layers. The sequence of strong pluvial phases centred around 124, 100 and 80 ka BP is confirmed by data and model results of precipitation and vegetation changes in the Sahel and Sahara [2] and by changes of the East African [77] and West African [78] monsoon intensities. African lake-level records provide a less consistent picture but generally indicate wet periods between 145–120, 110–95 and 80–65 ka BP (summary in [67]), which is more or less consistent with the marine and model data. These orbitally paced favourable phases likely provided recurrent time windows for migration pathways of modern humans out of Africa across the Sahara [67, 79, 80] confirming similar findings for the Arabian Peninsula [81–83]. According to our data, the strongest humid period occurred during the Eemian and was followed by two pronounced but less intense phases, which are centred at ca. 100 ka and ca. 80 ka. The subsequent dry phases are associated with fast lake-level drops and desiccation phases as indicated by erosional surfaces in lake successions, e.g. from lake Bosumtwi in Ghana [66] and from East African lakes [65, 67, 84].

The dust phase following sapropel S6 formation is weaker than one would expect based on the insolation and the monsoon index (Fig 6). This can be attributed to the generally cooler glacial boundary conditions, which dampen the intensity of African summer monsoon rainfalls [68], thus preventing the establishment of extensive lake and river systems and associated biomes. Changing wind systems cannot be responsible for the different patterns, because the regional wind direction and strength during glacial periods were not significantly different from that during interglacial periods [85].

### Heinrich Events

The generally low input of fine-grained dust to the EMS during the glacial periods was due to the limited availability of material following the major dust phases. However, during the time interval 65–11 ka the dust record was punctuated by a series of minor but abrupt K/C maxima correlating with Heinrich Events and other cold events of the North Atlantic and Arctic regions [19] (Figs 2 and 4). The Heinrich Events led to mega-droughts in the Sahel zone that are documented by enhanced dust deposition in the Atlantic Ocean off West Africa. This has been linked to a reduction of the Atlantic meridional overturning circulation and a corresponding southward shift of the African rain belt and drying of North Africa [3]. Our results extend the records of enhanced dust deposition in the western and easternmost Mediterranean Sea [69, 86] during Heinrich Events and suggest that such droughts also affected wide areas in the central and eastern parts of North Africa and dust delivery to the EMS due to stronger winds (Fig 5). Strong arid conditions during Heinrich Events are also documented by decreased Nile sediment discharge [15].

### Conclusions

We established a conceptual model of dust dynamics during late Quaternary humid and arid periods (Fig 5). During arid periods kaolinite deflation was restricted and mainly controlled by the aeolian erosion of kaolinite-bearing sedimentary rocks. During humid periods the aeolian transport of kaolinite ceased due to humidity, vegetation and reduced wind activity. Instead, kaolinite-bearing sedimentary rocks were fluvially eroded, and the clay-rich erosion products were stored in lake basins and soils. With the return of arid conditions the basins desiccated and the kaolinite-rich sediments and soils were blown out, leading to maximum kaolinite influx to the Eastern Mediterranean Sea. Intensified aeolian activity during glacial droughts (Heinrich Events) caused minor kaolinite maxima. The K/C data clearly show that the fine-grained dust fluxes are not only controlled by aridity but to a large extent by the availability of appropriate material from desiccated basins. In contrast to this material, dust generated by aeolian abrasion of sand grains, a major process in interior sand seas and alluvial fans (e.g. [41]), is more independent of rainfall and strongly related to wind intensity.

Our study has implications for estimating the monsoonal influence on North African environments and the dust export to the EMS. Vegetation-climate feedbacks led to abrupt regime changes and vegetation shifts at the end of the pluvial periods. We developed a dust index that quantifies the strength of preceding pluvial phases. Three humid phases during the last interglacial and early glacial time, with the strongest during the Eemian, provided corridors for the migration of modern humans to the Mediterranean region. Our data suggest that the present-day phase of dust export from former lake basins has not yet ended.
